# The complete mitochondrial genome of the *Minla cyanouroptera* (Passeriformes: Timaliidae)

**DOI:** 10.1080/23802359.2019.1677520

**Published:** 2019-10-16

**Authors:** Jie Huang, Chuang Zhou, Luyao Wang, Xue Jiang, Xiuyue Zhang, Bisong Yue, Yang Meng

**Affiliations:** a College of Biology and Food, Shangqiu Normal University, Shangqiu, Henan, China;; b Key Laboratory of Bio-resources and Eco-environment (Ministry of Education), College of Life Sciences, Sichuan University, Chengdu, Sichuan, China

**Keywords:** *Minla cyanouroptera*, mitochondrial genome, phylogenetic analysis

## Abstract

The blue-winged minla (*Minla cyanouroptera*) belongs to Timaliidae, which is a near threatened species in the IUCN Red List. In this study, the complete mitochondrial genome of *Minla cyanouroptera* was sequenced and characterized. The genome is 17,862 bases in length (GenBank accession no. MK940810). The overall base composition of *M. cyanouroptera* mitogenome is 30.9% for A, 31.5% for C, 13.8% for G, and 23.8% for T. The nucleotide sequence data of 13 protein-coding genes of 12 Passeriformes species were used for phylogenetic analyses. Trees constructed by using Bayesian and maximum-likelihood methods demonstrated that *Minla ignotincta* was closest to *M. cyanouroptera.*

The blue-winged minla (*Minla cyanouroptera*), also known as the blue-winged siva, is a species of bird in the Timaliidae, prefers to live in subtropical or tropical moist montane forests (Collar and Robson 2007). It is found in the Indian subcontinent and Southeast Asia, ranging across Bangladesh, Bhutan, Cambodia, India, Laos, Malaysia, Myanmar, Nepal, Thailand, Tibet, and Vietnam (Collar and Robson 2019). Although morphological (Yu and Guo 2007) or phylogenetic research based on the partial cytochrome b gene sequence (Luo et al. 2009) have been carried out in recent years, the complete mitochondrial data of *M. cyanouroptera* were still lacking.

In this study, we sequenced the complete mitochondrial genome of *M. cyanouroptera* (GenBank accession no. MK940810). The muscle sample was obtained from a wild *M. cyanouroptera* that died of natural causes in Laojunshan nature reserve, Sichuan Province, China (104°00.99′, 28°41.98′). The voucher specimen (SCGB00349) was deposited in Sichuan University Museum, Chengdu, China. The complete mitochondrial genome sequence of *M. cyanouroptera* was amplified and sequenced by 20 pairs of primers using normal PCR methods.

Similar to other Timaliidae mitogenomes, the complete mitochondrial genome of the *M. cyanouroptera*, with a length of 17,862 bp, contains 22 tRNA genes, 13 typical protein-coding genes, 2 rRNA genes (*12S rRNA* and *16SrRNA*), and two control region (D-loop1 and D-loop2) (Zhou et al. 2015; Li et al. 2016; Zhang et al. 2016;). The base composition of mtDNA is 30.9% A, 23.8% T, 31.5% C, and 13.8% G, so the percentage of A + T (54.7%) was slightly higher than G + C (45.3%). Among 13 PCGs, the shortest one was *ATP8* gene (168 bp) and the longest one was the *ND5* gene (1818 bp). All PCGs began with ATG. Of the 13 PCGs, 11 used complete (TAA) or incomplete (TA or T) stop codon, while *ND5* and *COI* ended with AGA and AGG, respectively. One of the 13 PCGs (*ND6*) encoded on the L-strand while others encoded on the H-strand. The inferred secondary structures of 21 tRNAs (excluding tRNASer (AGY)) of *M. cyanouroptera* were all conformed to the common structural features of mitochondrial tRNAs. There were eight tRNA genes (*tRNAGln*, *tRNAAla*, *tRNAAsn*, *tRNACys*, *tRNATyr*, *tRNASer (UCN)*, *tRNAPro*, and *tRNAGlu*) encoded on the L-strand.

We used the maximum-likelihood (ML) and Bayesian methods based on the 13 protein-coding genes to analyze the phylogenetic position of *M. cyanouroptera* (Figure 1). The parameters and methods to build the two different phylogenetic trees were followed Li et al. (2016) and Liu et al. (2019). As shown in Figure 1, the different tree-building methods yielded the same topology. *Minla cyanouroptera* is clustered with *Minla ignotincta*, which indicates that they have a much closer relationship (Dickinson and Christidis 2003). The species of genus *Minla* was the sister lineage to the clade formed by Leiothrix with both strong bootstrap value and posterior probability, which was consistent with the previous molecular evidence (Li et al. 2016). The present study first reported complete mitochondrial genome of *M. cyanouroptera* and we believed that the mitogenome data will contribute to the evolutionary analysis of Timaliidae.

**Figure 1.**
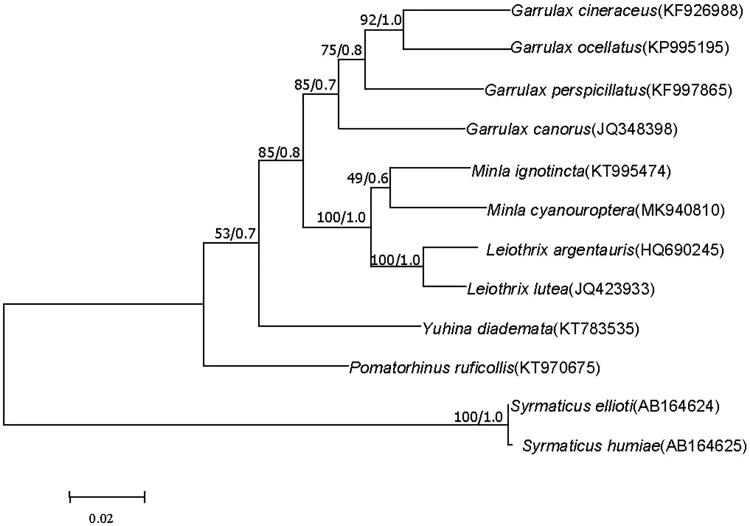
Maximum-likelihood (ML) and Bayesian tree based on combining 13 mitochondrial protein-coding gene sequences of 10 Timaliidae and 2 Syrmaticus birds. The Syrmaticus birds were used as the outgroup. ML bootstrap values/Bayesian posterior probabilities are shown above nodes.
